# Mixed-Method Evaluation of the Impact of a “Healthcare Exploration Through Patient Care” Course on Undergraduate Students

**DOI:** 10.1007/s40670-025-02545-y

**Published:** 2025-11-08

**Authors:** Jacqueline M. Knapke, Joshua Peter, Arthur Pancioli, Alex Maus, Deana Brown, Deanna Markus, Travis Scarfpin, Valerie Hoagland-Scarfpin, Kate Carey, Anil Menon

**Affiliations:** 1 Department of Family and Community Medicine, University of Cincinnati, Cincinnati, OH USA; 2 Department of Molecular and Cellular Biosciences, University of Cincinnati, Cincinnati, OH USA; 3 Department of Emergency Medicine, University of Cincinnati, Cincinnati, OH USA; 4 Educational Placements, UC Health, Cincinnati, OH USA; 5 Human Resources, UES Inc., Dayton, OH USA; 6 College of Medicine, University of Cincinnati, Cincinnati, OH USA; 7 University of Cincinnati College of Medicine, Medical Sciences Building 4453-B, PO Box 670582, Cincinnati, OH 45267-0582 USA

**Keywords:** Undergraduate education, Healthcare careers, Internship, Clinical experience

## Abstract

**Introduction:**

Students in undergraduate degree programs often do not gain exposure to clinical work until late in their studies or even after graduation. The Healthcare Explorers (HCE) program provides an introduction to healthcare careers via a course series that combines seminar speakers and paid internships for students as patient care assistants. The purpose of this study was to evaluate the effectiveness of the gateway course in the HCE series using student feedback and reflection data.

**Methods:**

Mixed-method data were collected from students through feedback surveys and written reflection assignments. Descriptive statistical analyses described frequencies for the quantitative survey items. Inductive qualitative analyses identified themes that describe the impact of the course over time on students’ perceptions, understanding, and decisions regarding future careers in healthcare, as well as areas of program improvement.

**Results:**

Quantitative results demonstrated consistently high satisfaction throughout the semester and between semesters. Several themes characterized students’ self-reflexivity in the written reflection data: feelings of inspiration and relief, consideration of personal and professional goals, self-confidence in skills and character, awareness of career options, and key lessons learned.

**Discussion:**

Introducing students to patient care, clinical team roles, and career options early in their education prepares them to pursue the training and work experiences they need to achieve their individual goals. Formal programming such as the HCE program that combines a supportive course framework with a clinical internship provides students with critical experiences that encourage self-reflection and informed decision-making.

**Supplementary Information:**

The online version contains supplementary material available at 10.1007/s40670-025-02545-y.

## Introduction

Gaining hands-on clinical experience is a crucial component of healthcare education, yet many undergraduate students in undergraduate degree programs do not have the opportunity to engage in clinical work until the later stages of their education. The importance of early clinical exposure has grown in recent years, with professional schools increasingly expecting direct patient care experience [1]. Without this, students may struggle to understand the demands of healthcare professions, leading to uncertainty in career paths and potential dissatisfaction after a very long course of education.

In the increasingly competitive landscape of admissions to medical and other professional healthcare schools, applicants who lack direct clinical experience are often at a disadvantage even if they possess strong academic records and extracurricular involvement [2]. Admissions committees now place greater emphasis on demonstrated patient care experience as a measure of an applicant’s readiness for a career in healthcare [3]. Without this background, even high-achieving students may be overlooked in favor of applicants with patient experience. This trend demonstrates a change in admissions priorities, in alignment with the pre-medical competencies list from the Association of American Medical Colleges (AAMC), which includes competencies such as service orientation, social skills, and cultural humility [4]. Competencies of this nature are best demonstrated in applicants with experience in patient interaction.

In addition to hands-on clinical experience, many undergraduate students lack an awareness of the vast array of career paths and specialties within healthcare. This deficit in knowledge can lead to students completing medical school and second guessing their career choice. Providing students with clinical experience and diverse perspectives can significantly enhance their understanding of their career path and strengthen their professional school applications [5]. Students who gain hands-on exposure through volunteering, paid positions, and shadowing develop deeper insight into their chosen field, better preparing them for their future careers [5, 6].

Despite this newly emerging emphasis on clinical experience and the benefits it provides, many undergraduate students face barriers to obtaining it [7]. Without degrees or certificates such as a State Tested Nurse Aid or medical assistant, the clinical experiences open to aspiring healthcare professionals are very limited. Most of these certifications must be completed outside of a student’s home university and often require enrollment in external programs. These programs can be both time-consuming and costly, posing significant logistical barriers for students already balancing full academic course loads.

Few studies have examined the impact of exposure to clinical care through formal undergraduate coursework. One study incorporated an asynchronous module focused on increasing undergraduate students’ understanding of the path to medical school into an introductory biology research skills course. Using a pre- and post-evaluation survey methodology, they found a 28.7% increase in students’ reported understanding of pre-health requirements [8]. Another study explored how viewing and coding video-recorded patient encounters impacted pre-medical students who were then asked to collect new patient histories on a standardized patient, pre- and post-encounter review. This study found that students spent more time taking a history and asked more questions after observing a recorded patient encounter; students also reported in follow-up interviews that viewing and coding the recorded patient encounters was transformative to their understanding and envisioning their future selves as a clinician [9]. While these studies provided students with useful foundational experiences to better prepare them for healthcare careers, they did not provide students with longitudinal, direct patient care experiences. Another study went one step further by providing undergraduate students with didactic training, active learning activities, and structured shadowing experiences with clinicians, finding that students gained insights and exhibited greater confidence and understanding of healthcare compared to a control group [10]. Students in this program shadowed for 2 h a week over 10 weeks, rotating among different specialties.

Recognizing an urgent need for support staff in the local healthcare workforce and a large undergraduate population of students who could help fill those roles while also gaining critical experience to better prepare them for careers in nursing and medicine, the Healthcare Explorers (HCE) program was founded in 2021 as a collaborative program between the University of Cincinnati (UC) College of Medicine (COM) and UC Health, which operates the University of Cincinnati Medical Center (UCMC) [11]. Following the “Co-op 2.0” model endorsed by UC [12], the HCE program provides a critical introduction to careers in healthcare via a course series that combines seminar speakers and longitudinal paid internships for students as patient care assistants (PCAs) on clinical teams in the acute care units at UCMC. The purpose of this study was to evaluate the effectiveness of the gateway course in the HCE series, using mixed-method feedback and reflections provided by students.

## Materials and Methods

### Setting and Context

The HCE course series is comprised of six elective courses open to students from all types of degree programs at UC. In order to be eligible for participation in the program, applicants must be current UC students who have completed at least 1 year of undergraduate study and maintained a minimum 2.5 grade point average on a 4.0 scale. “Healthcare Exploration through Patient Care 1” is the gateway course in the series; it is offered every fall and spring semester and requires in-person attendance at a weekly seminar as well as completion of 168 in-person work hours as PCAs at UCMC. The weekly seminar introduces several topics, including development of a high-impact personal statement, creation of an effective resume, and introduction to different career pathways within healthcare via guest presenters each week. A sample seminar schedule is provided in Table 1. Students are also required to submit several written assignments: brief, weekly reflection papers, a resume, and a personal statement. After successfully completing the gateway course, students can go on to sign up for five follow-up courses in order to continue their PCA internships longitudinally.

**Table 1:** Sample seminar schedule for the HCE gateway course*

Week	Topic
1	Welcome, Course Objectives, and Overview of Syllabus
2	Rehabilitation Services (Physical Therapy & Occupational Therapy) Nursing Careers
3	Resume Workshop Physician Assistant Career Pathways
4	Journey to Trauma Surgery Air Care/Mobile Care Professional Careers
5	Careers in Cardiology (MD and RN) Journey to Becoming a Nurse Practitioner
6	Clinical and Translational Research Careers Working with a Diverse Patient Population and Workforce
7	Crafting a Good Personal Statement Pharmacy Clinical Practice
8	LGBTQIA + Primary Care LGBTQIA + Occupational Therapy
9	Professional Etiquette and Mock Interviews Careers in Radiology
10	Journey to Becoming a Respiratory Therapist Careers in Genetic Counseling
11	Careers in Psychiatry Medical Student Panel
12	Journey to Gynecological Oncology Careers in Speech Language Pathology
13	Careers as a Certified Anesthesiologist Assistant/Certified Registered Nurse Anesthetist (CAA/CRNA) Addiction Medicine Careers
14	Semester Wrap-Up and Feedback Session

*In addition to attending at least 10/14 seminars, students submitted weekly written assignments including brief reflection papers, a resume, and a personal statement. Students were also required to work an average of 12 h per week as a PCA at UCMC

As part of the gateway course requirements, students are expected to complete 12 h of hospital-based experiential learning each week. To align with the operational needs of the hospital, at least half of these hours must be fulfilled during off-shift times, such as evenings, nights, or weekends. Students apply for the course through an online application and participate in a phone interview to assess fit and readiness. Once approved, they become employees of UC Health and complete a 2-week orientation program, which introduces them to roles in healthcare and resources and services available within UCMC, while also providing training in topics such as professionalism, communication, signs and symptoms, clinical measurements, the electronic health record, specimen collection, de-escalation techniques, and patient documentation.

As part of the selection process, applicants rank their top three preferred clinical units from a diverse list that includes women’s health, cardiology, neurology, psychology, internal medicine, surgery, pharmacy, transportation, intensive care (ICU), emergency medicine, laboratory, radiology, respiratory therapy, ambulatory care, rehabilitation, and clinical research. Placement decisions are made based on student preferences, application materials, and hospital needs. Once placed, students take on a range of responsibilities that may include checking vital signs, charting patient inputs and outputs, assisting patients with ambulation and meals, bathing patients, and performing blood glucose checks. On units that have patients requiring specialized or intensive care, such as psychiatry, HCEs take on additional rehabilitation-focused responsibilities. These may include facilitating activities like art and music therapy. Students’ clinical performance is evaluated several times throughout the semester by hospital staff, including nurses and nurse managers, using a feedback survey accessed by a QR code located on each student’s badge.

### Study Design

This evaluation study combines quantitative and qualitative methods. Data were collected over three academic years, or six semesters: beginning with course inception in fall semester 2022 and ending in spring 2025. The quantitative data and some qualitative data were collected via two surveys each semester: one that was administered at the midpoint of each semester and a second that was administered at the end of each semester. Both surveys were administered to students using Microsoft Forms via Canvas. The mid-semester survey included one item with a Likert response scale of agreement and two open-ended questions. The end-of-semester survey included twelve Likert-scaled items and three open-ended questions. For all Likert-scaled items, the scale was 1: Strongly Disagree, 2: Disagree, 3: Neutral, 4: Agree, and 5: Strongly Agree. Both surveys are provided in supplemental Appendix A. Additionally, student reflections that were collected as course assignments were included in the qualitative dataset: we collated the first (pre) and last (post) reflection papers for each student enrolled in the HCE gateway course during the study time frame.

Descriptive statistical analyses were conducted to describe frequencies for the quantitative survey items. Since neutral responses often reflect a lack of meaningful agreement, all neutral responses (scored 3 on the Likert scale) were dropped when calculating mean scores, effectively transforming the 5-point scale to a 4-point scale [13]. Pre-reflection papers and post-reflection papers were first analyzed as two datasets in order to effectively compare themes from two time points. We conducted an inductive, phenomenological qualitative analysis on the pre- and post-reflection papers. Data were analyzed for themes to distill the key facets of the educational experience and to identify themes that describe the impact of the course over time on students’ perceptions, understanding, and decisions regarding future careers in healthcare [14]. Qualitative analysis of the pre- and post-reflections focused on themes around students’ self-reflexivity in an effort to understand if they had grown over the semester and how. In the inductive qualitative analysis, we did not include codes or themes that related to specific course content (e.g., suturing practice), but rather sought to characterize students’ perceptions and growth more generally as they explored their career pathways as individuals. Qualitative data from the mid-semester and end-of-semester surveys were analyzed separately for themes identifying areas of improvement. This was a retrospective, mixed-method evaluation study aimed at program improvement. The protocol was reviewed by the UC Institutional Review Board (IRB) and determined to be “not human subjects” research (IRB# 2024-0522). Additionally, the protocol received an ancillary Family Educational Rights and Privacy Act (FERPA) review by the UC Office of General Counsel.

## Results

### Mid-Semester Evaluation Survey Data: Quantitative Results

**Fig. 1:**
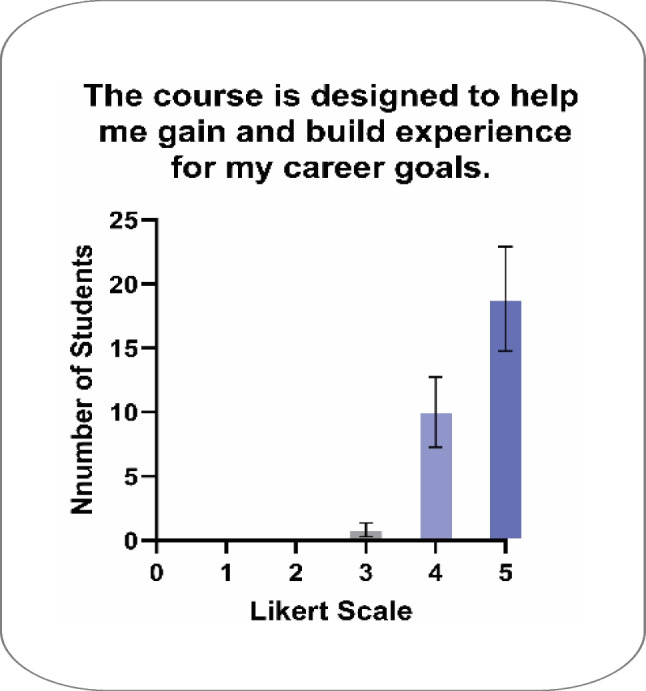
Students express satisfaction from mid-semester course survey. Shows the mean responses (1—strongly disagree, 2—disagree, 3—neutral, 4—agree, and 5—strongly agree) for each semester’s mid-semester survey. ± SEM. *N* = 171 for all semesters and *n* = 20, *n* = 36, *n* = 51, *n* = 38, *n* = 14, and *n* = 12 for Fall 2022, Spring 2023, Fall 2023, Spring 2024, Fall 2024, and Spring 2025 semesters respectively

**Fig. 2:**
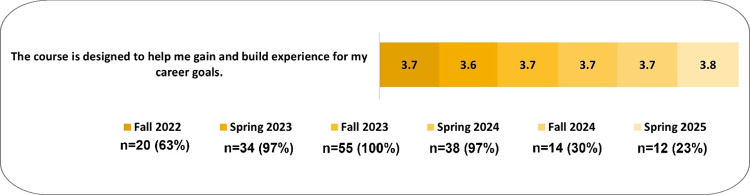
Satisfaction of the course was demonstrated consistently between semesters. Shows the mean results of the mid-semester survey on a 1–4 scale with 1—strongly disagree, 2—disagree, 3—agree, and 4—strongly agree. A neutral response was given as an option but was removed from this mean data. Neutral responses represented 3% of responses overall

Between fall 2022 and spring 2025, 258 students enrolled in the HCE gateway course. One hundred and seventy-one students completed mid-semester evaluation surveys for an overall response rate of 66%. Analysis of responses to the single Likert-scaled question indicated high levels of satisfaction with the course design supporting students’ gaining experience for their career goals (Fig. 1), with satisfaction remaining consistent between semesters (Fig. 2). Response rates varied by semester; Fig. 2 provides both the number received and the response rate.

### Mid-Semester Evaluation Survey Data: Qualitative Results

Analysis of responses to the open-ended questions in the mid-semester survey determined two themes within the first question of what students liked or enjoyed most: hands-on patient care experiences they had within the internship component of the course, and the wide array of speakers who shared their journeys within the seminar component of the course. Themes in responses to the second open-ended question on the mid-semester survey about how to improve the course varied as the course evolved over time. For example, in the first semester, students described the difficulty of meeting the minimum work hour requirement (18 h per week) and the variability of the seminar schedule. Both of these issues were addressed in the following semester, so the themes in the subsequent three semesters included: more interactivity during the seminar, better organization of internship and seminar requirements, and a shorter seminar time. To address these issues, the core teaching team began to encourage guest speakers to build more interactivity into their presentations; we developed a comprehensive syllabus that described internship and seminar requirements clearly and discussed the syllabus in detail in the first class each semester. We could not change the length of the seminar (2.5 h) in order to meet minimum requirements for the number of credits students receive for completing the course.

### End of Semester Evaluation Survey Data: Quantitative Results

**Fig. 3:**
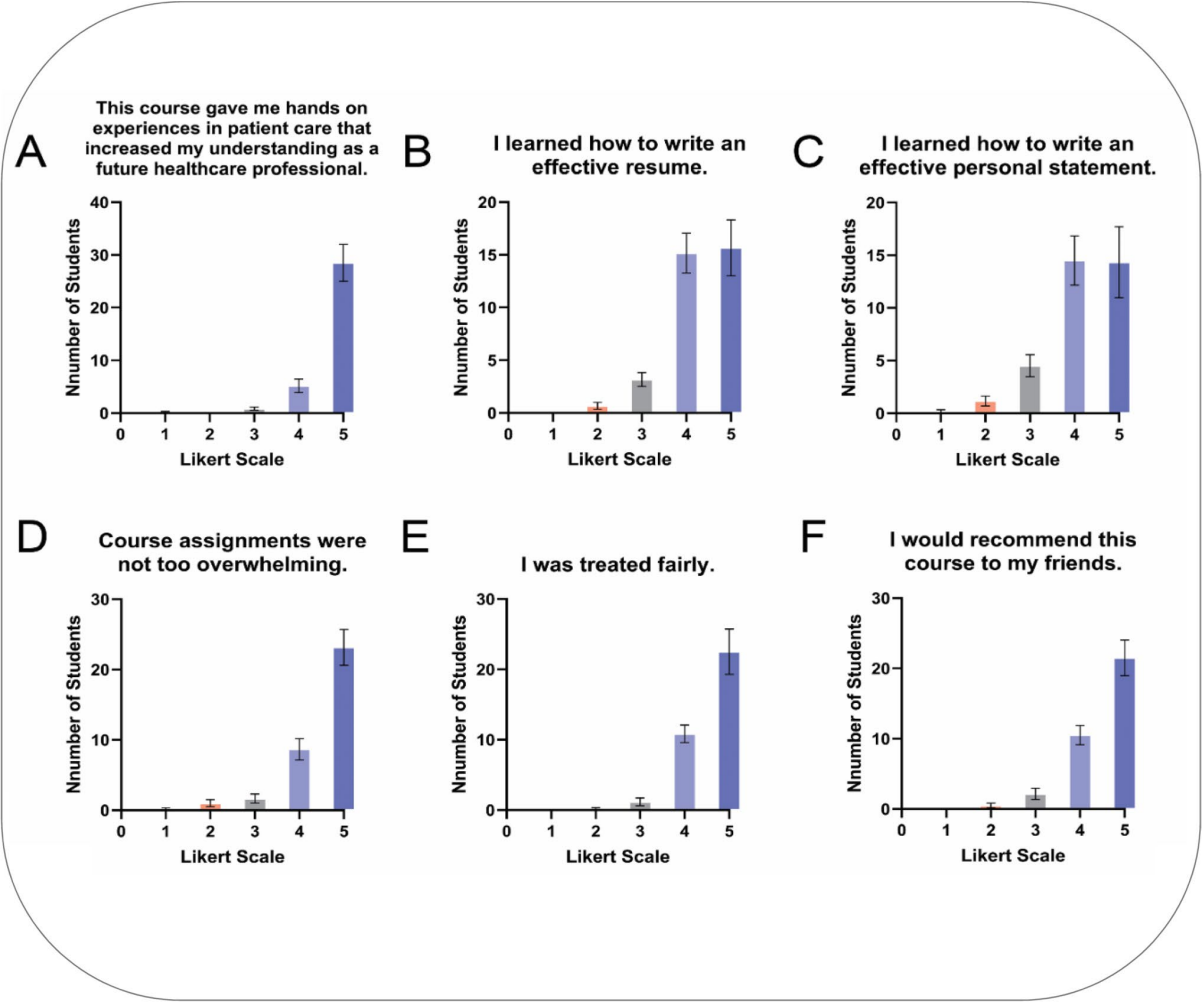
Students express satisfaction from end-of-semester course survey. Shows the mean responses (1—strongly disagree, 2—disagree, 3—neutral, 4—agree, and 5—strongly agree) for each semester’s end-of-semester survey questions. ± SEM. *N* = 208 across all semesters and *n* = 30, *n* = 35, *n* = 47, *n* = 47, *n* = 38, *n* = 42, and *n* = 16 for Fall 2022, Spring 2023, Fall 2023, Spring 2024, Fall 2024, and Spring 2025 semesters respectively

**Fig. 4:**
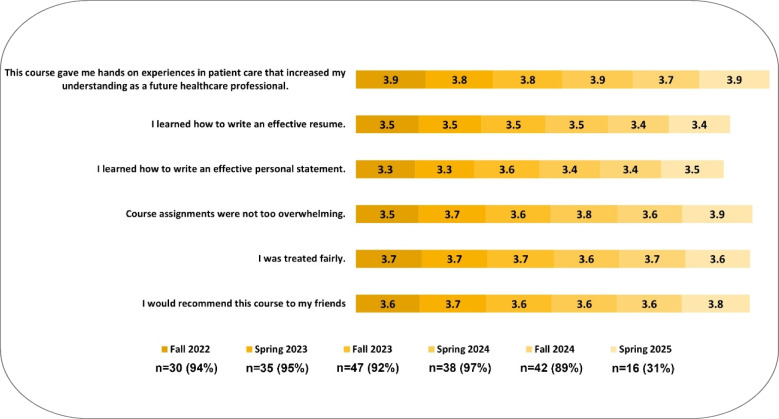
Students consistently express satisfaction with the course across all semesters. Shows the mean score of student responses to the questions asked in the end-of-semester course survey. Scale is 1–4 (1—strongly disagree, 2—disagree, 3—agree, 4—strongly agree) with neutral responses having been dropped. Neutral responses represented 6% of responses overall

Between fall 2022 and spring 2025, 208 students completed end-of-semester evaluation surveys for a response rate of 80%. End-of-semester survey results demonstrate high levels of satisfaction across all survey items (Fig. 3), and consistency across 3 years of student feedback (Fig. 4).

In particular, students strongly agreed that the course gave them hands-on experiences in patient care that increased their understanding, and that they learned how to give and receive feedback in a constructive way.

Using student feedback, we have made several other small changes to the course as well. For example, the work hour requirement started at 18 h per week but was dropped to 12 after students suggested 18 was more than they could complete. Hospital units in which students can rotate have been added over time based on student interests. In the course’s current iteration, we leverage the experiences of a learning assistant—an undergraduate who has completed the HCE program and moved into a PCA position—by introducing a presentation called “HCE Tips” to the weekly seminar. The HCE Tips are focused on foundational, practical aspects of being an HCE, such as the different types of work and patients students can expect to find on different units, important locations such as nursing operations, food services, and sterile processing, reminders for ensuring Health Insurance Portability and Accountability Act (HIPAA) guidelines are followed, and tips for serving as a patient sitter.

### End of Semester Evaluation Survey Data: Qualitative Results

When asked to provide constructive feedback for the teaching team in the end-of-semester survey, analysis of responses to this open-ended question provided several consistent suggestions for improvement. In the first two semesters, students noted the lack of seminar presenters from racial or ethnic minorities and from fields outside of medicine (e.g., nursing or other healthcare careers), and they requested more diversity in speakers’ identities and disciplines. We addressed this issue by including more non-physician or nurse roles, such as physical therapy, occupational therapy, respiratory therapy, clinical research, and speech-language pathology. We also intentionally invited speakers who represented more diverse demographics. Students also suggested more seminar speakers who could help students navigate upcoming milestones, such as preparing for the Medical College Admission Test (MCAT), applying to medical or professional school, and funding advanced degrees, so we introduced a medical student panel that includes medical students of all years to share their experiences in preparing for and succeeding in medical school. Finally, some students suggested better coordination and response time from the teaching team, particularly with regard to the internship. We addressed this issue by initiating regular teaching team meetings and prioritizing email responses to students so that their questions were answered in a timely manner.

### Qualitative Student Reflection Data Results

In addition to the survey data, students submitted 252 reflections as their first assignment (98% of the total number of students) and 200 submitted final reflection assignments (78% of all students). The drop in submission rate for the final reflection paper is likely due to the fact that within the written assignment requirement for the course, students are required to submit at least ten reflection papers during the 14-week semester. For many, if they have already submitted their ten reflections, they do not submit the last one. Using reflection data, we identified several themes that characterized students’ self-reflexivity as they attempted to connect course experiences from both their internship and from course guest speakers to their own lives and goals. In the pre-reflection data, students expressed feeling inspired to overcome future challenges, seize opportunities to better understand themselves and build confidence, and pursue their passions—including taking chances that might make them uncomfortable. They also expressed relief that they have time to figure out their individual journeys and that the speakers’ career paths were remarkably nonlinear, so students should take their time, keep an open mind, embrace flexibility/adaptability, and enjoy the ride. Two themes carried from pre-reflection data into post-reflection data: reflections on lessons learned, and a discussion of individual goals, with an emphasis on considering the personal factors that will affect their professional decisions. Unique themes found in post-reflection data included: self-confidence in their skills and character and awareness of the career options available to them—the majority expressed a commitment to healthcare careers, but they were more open-minded about non-clinical routes such as healthcare administration. Table 2 provides a summary of thematic findings, including representative quotations for each theme.

**Table 2:** Summary of qualitative themes from student reflection papers

Theme	Brief description	Pre- or post-data	Representative quotations
Inspiration	Feeling inspired to: · Demonstrate fearlessness and determination to overcome challenges · Seize every opportunity that comes their way · Pursue passions and take chances	Pre	“The lesson that I found most important was to take the opportunities presented to you. [The presenter] took every new opportunity and faced it fearlessly. I am someone who fears the unknown. [The presenter] made me realize that no one decision can define a lifetime.” “... I can be so confident about what I think my life will look like, but changing plans is okay. I am a huge planner, I like to have a plan A, B, and C for every aspect of my life. Hearing about how [the presenter’s] plans changed, how she took on new roles that she never envisioned herself in, and how she ended up loving them was really inspiring. I think moving forward, I need to have a more open mind about my future and not restrict myself to simply what I have planned for myself.”
Relief	Feeling a sense of relief that: · They have time to figure out their individual journeys · Career paths are often nonlinear · They can keep an open mind about their career options	Pre	“When our speaker told us about her change in path which worked out for her I felt relieved because I was hearing from an other person that the road doesn’t always have to be straight cut.” “I have also been struggling to choose between [two specific healthcare career tracks], so it’s relieving to know that I am not the only one thinking about both routes. In my classes, it can get very intimidating to see that everyone has a set goal for their future. From [the presenter’s] talk, it was nice to be reminded that everyone is on their own individual path and that I have resources to reach out to mentors for advice.”
Life goals	Reflecting on their goals, both personal and professional, with an emphasis on the financial or family factors that will impact their decisions	Pre	“Something that I really connected with [the presenter on] was also a goal of becoming a mother. I know that for a while I wanted to be an anesthesiologist but that has changed because of wanting a family.” “Ever since I set my heart on developing a career in the healthcare field I prepared myself to take the standard pathway, majoring in a science course, volunteering and doing some research along the way, finding meaningful experiences, studying for the MCAT, taking it, and going to medical school. However, now as a second year biological sciences student I have been wondering if that is the correct path for me... I shared some similarities with the speaker, she mentioned being family orientated with a heavy focus in being a mother. This is a sentiment that I also share although I am young.”
		Post	“I will seriously consider joining this [federal] program if I get accepted to medical school however I might not after thinking of my family as well. I’m going to be away for medical school studying with little time to see them, my family is my support and without that it would make studying much more difficult.” “I would love to join the [program presented] and have thought about it a lot. Unfortunately, I have a lot of physical and mental health issues that won’t permit me to join. I do plan to do medical volunteering outside and inside of the country once I am a doctor. I will still be able to give back just in an alternate way.”
Lessons learned	Describing high-level lessons from the semester, such as the importance of teamwork and empathy when working in a clinical setting	Pre	“You need to have a heart to be in healthcare. You should have emotions and care about your work. If you do not care, then you should not have a career in healthcare.” “The most important thing [the presenter] taught me was that regardless of which position you hold in healthcare your whole world pauses when you enter the room of a patient. This is so insightful and definitely an ideal that I will hold close for the rest of my life.” “... success is not simply the result of the luck/chances we have in life but rather it is the decisions we make regarding the chances we’ve been given that can lead to our success. Related to that, those decisions should be made not based on fear but rather on the forward-thinking considerations we have made about what we want for our futures.”
		Post	“[The presenters’] insights and experiences provided a new perspective on leadership, teamwork, and problem-solving, which are applicable... in other aspects of life... I learned about the importance of discipline, adaptability, and perseverance in achieving success. The... emphasis on teamwork and the idea of the whole being greater than the sum of its parts was also an important takeaway. It highlighted the importance of working together towards a common goal and the value of collaboration in achieving success.”
Increased self-confidence	Expressing greater confidence in their skills, their character, and their potential for success in healthcare	Post	“I was quite afraid of being on a new floor... I was very fortunate that when the family came in they could see how cheerful I was to take care of their family member. I got to talk with the patient and their family members... I learned a lot from them and found that I was the only person on the floor past and present who really wanted to take care of them and connect with them. I truly felt that I made a positive impact because I wasn’t afraid to do my job and engage with the people in the room in a positive way for several hours!” “I received a very heartfelt compliment from the patient and his wife that I was doing an amazing job and that I made them feel more comfortable than any other caretaker at the hospital, thus far into their stay... This shows that the work I’ve put in on improving my quality of care this semester has highly improved from the start.”
Increased awareness of career options	Describing healthcare career pathways that are available to them, including non-clinical options	Post	“Hearing about [an organization’s] offered programs also gives you a lot to think about. The benefits are really incredible. I wouldn’t mind free medical school as well as a substantial paycheck while in residency. I’ve even met nurses and residents [from this organization] who have told me about what the [program] offers. It all definitely gives me something to think about.” “As someone who is fairly sure they want to go to medical school, but not dead set, it was refreshing to hear [the presenter’s] story. I also feel like I learned that there are significant opportunities for jobs despite having gone to a specific graduate program... This opened my eyes to the fact that even if I go to medical school, I have a little less cause for stress and concern, as there are a wealth of other healthcare related opportunities regardless of degree.”

## Discussion

Undergraduate students who are interested in healthcare careers are frequently only aware of physician or nurse career pathways because those are the roles they have had the most interactions with in the past. They may have an inherent interest in science or a desire to care for others, but they often begin applying for medical or other professional schools without hands-on, longitudinal internships in clinical care and with limited exposure to other healthcare careers. The HCE program at UC/UC Health fills a critical gap in experience for undergraduate students by providing extended, applied experiences working in a hospital setting while also introducing them to a variety of medical specialties and other healthcare careers in a weekly seminar. Study results demonstrate high student satisfaction over three years, particularly with helping students gain experience to achieve their career goals (3.68 mean score in mid-term evaluation and 3.83 mean score in end-of-semester evaluation on an adjusted 4.0 scale). Similarly, thematic findings demonstrate increased self-confidence and awareness of career options in the post-reflection qualitative data.

In our experience, students have been able to manage work hour requirements with seminar attendance and written assignments. Assignments are intended to be low-stakes and practical, encouraging students to reflect on their experiences but also take steps towards developing a strong resume and personal statement for professional or graduate school applications. This study provides further evidence for the importance of providing undergraduates with experiences working in healthcare and early exposure to healthcare careers. One recent collective case study summarized how college students prepare for medical school concluded:

There remains a gap in the educational process that should inform students about careers and healthcare.... there is also a need to guide students to other careers in health

and suggests that specific coursework about medicine and early exposure to alternate career pathways in healthcare is needed [15]. The gateway course in the HCE program is one way to address that critical gap. A recent study evaluating the effect of an undergraduate healthcare experience course on students found that student participants demonstrated a high interest in science and healthcare, particularly rural health, attained novel learning and insights from the experience, and demonstrated higher confidence and a significant increase in understanding of healthcare when compared with a control group of non-participant students [10]. Qualitative findings from our study echo these results, affirming the transformative potential that experiences such as the HCE program offer for undergraduate students. Interestingly, a new theoretical framework suggests that career literacy, or the ability to “understand, interpret, evaluate, and make decisions related to career information” is as important as academic literacy to student success [16]. Two key components of career literacy are self-efficacy and career experiences, components that can be supported during undergraduate studies by initiatives like the HCE program.

This study has several strengths, including using student feedback to inform program evaluation longitudinally and making small changes to improve the program over time. Students have the opportunity to work in a large variety of units (currently 16) in the hospital to gain different experiences that help them understand different team roles across different medical disciplines so they can make better-informed decisions about their own career pathways. However, it also has some limitations. Cohorts self-select based on a strong interest in gaining admission to medical or other health careers, and therefore, the opinions provided via feedback mechanisms could be considered biased, as students may be expressing high satisfaction due to the perceived advantages they receive from participating in the program, rather than the program itself. Relatedly, while student satisfaction data can be a useful metric to evaluate student experience, satisfaction data are also limited in their usefulness because students are often not the best judges of their own learning. This study includes a fairly small sample at one university, so results may not be generalizable to a larger sample in a different context. Additionally, response rates to the voluntary evaluation surveys varied between mid-semester, end-of-semester, and cohorts, so survey feedback may not be representative of the sample as a whole. Student experiences were limited to acute care units at an urban, safety-net hospital, which is unique from rural, suburban, private, or other types of healthcare settings. However, students were able to move between units during their internship in order to gain a variety of experiences within the hospital, and additional units were added as the program evolved. UCMC is the only Level 1 trauma center in the region, which also affects student experiences, as the patients they care for are often critically ill. Exposure to this level of care could be viewed as a strength or a limitation, as it introduces students to the full capability of contemporary medicine, but it may impact their career decisions if the environment is not a good match with their strengths and interests.

The HCE program at UC/UC Health is an innovative program that provides students with impactful clinical and educational experiences that inform their career decisions. Mixed-method feedback and reflections provided by students demonstrate the positive impact the gateway course had on student learning, confidence, awareness of career options, and personal and professional development. Future studies are needed to determine the impact of clinical work experiences and career pathway exposure on eventual student career choice. Student career outcomes may be explored using follow-up survey data and/or LinkedIn data collected from student profiles.

## Supplementary Information

Below is the link to the electronic supplementary material.

ESM 1 (DOCX 23.1 KB)

## Data Availability

The datasets generated and analyzed for this study are available from the corresponding author on reasonable request.
