# Supplementary material for: Mixed-Method Evaluation of the Impact of a “Healthcare Exploration Through Patient Care” Course on Undergraduate Students
Source: Med Sci Educ. 2025 Nov 8;36(1):213–23. doi: 10.1007/s40670-025-02545-y (PMC13043977; doi:10.1007/s40670-025-02545-y)
Supplement: Supplementary file 1 — (DOCX 23.1 KB) [file 40670_2025_2545_MOESM1_ESM.docx]

**Appendix A: Mid-Semester and End-of-Semester Evaluation Surveys**

**Mid-Semester Evaluation Survey**

When you submit this form, it will not automatically collect your details like name and email address unless you provide it yourself.

| **Question** | **Strongly Agree** | **Agree** | **Neutral** | **Disagree** | **Strongly Disagree** |
| --- | --- | --- | --- | --- | --- |
| 1. The course is designed to help me gain and build experience for my career goals. |  |  |  |  |  |

1. What do you like/enjoy about the course?
2. What would you like to see improved in the course?

**End-of-Semester Evaluation Survey**

When you submit this form, it will not automatically collect your details like name and email address unless you provide it yourself.

| **Question** | **Strongly Agree** | **Agree** | **Neutral** | **Disagree** | **Strongly Disagree** |
| --- | --- | --- | --- | --- | --- |
| 1. This course gave me hands on experiences in patient care that increased my understanding as a future healthcare professional. |  |  |  |  |  |
| 1. I learned how to give and receive feedback in a constructive way. |  |  |  |  |  |
| 1. I learned how to write an effective resume. |  |  |  |  |  |
| 1. I learned how to write an effective personal statement. |  |  |  |  |  |
| 1. I used the guided journal to reflect on my experiences in the hospital to develop effective habits. |  |  |  |  |  |
| 1. Course assignments were not too overwhelming. |  |  |  |  |  |
| 1. [Graduate teaching assistant name] was approachable and an effective Teaching Assistant. |  |  |  |  |  |
| 1. [UCMC HCE team member names] were approachable and effective organizers for my hospital experiences. |  |  |  |  |  |
| 1. The course director and team listened to my feedback. |  |  |  |  |  |
| 1. I was treated fairly. 2. I plan to enroll for MEDS 3100. |  |  |  |  |  |
| 1. I would recommend this course to my friends. |  |  |  |  |  |

1. Any constructive suggestions for the MEDS 3099 team?
2. Any particular requests for MEDS 3100?
3. My favorite speaker was?
